# Lifestyle precision medicine: the next generation in type 2 diabetes prevention?

**DOI:** 10.1186/s12916-017-0938-x

**Published:** 2017-09-22

**Authors:** Pascal M. Mutie, Giuseppe N. Giordano, Paul W. Franks

**Affiliations:** 1 Department of Clinical Sciences, Genetic and Molecular Epidemiology Unit, Lund University, Skåne University Hospital, SE-205 02 Malmö, Sweden; 2 Department of Public Health & Clinical Medicine, Umeå University, Umeå, Sweden; 3 Department of Nutrition, Harvard School of Public Health, Boston, MA USA; 4 Oxford Center for Diabetes, Endocrinology & Metabolism, Radcliff Department of Medicine, University of Oxford, Oxford, UK

**Keywords:** Review, Type 2 diabetes, Lifestyle factors, Overweight/obesity, Precision medicine, Biomarkers, Intervention, Prevention

## Abstract

The driving force behind the current global type 2 diabetes epidemic is insulin resistance in overweight and obese individuals. Dietary factors, physical inactivity, and sedentary behaviors are the major modifiable risk factors for obesity. Nevertheless, many overweight/obese people do not develop diabetes and lifestyle interventions focused on weight loss and diabetes prevention are often ineffective. Traditionally, chronically elevated blood glucose concentrations have been the hallmark of diabetes; however, many individuals will either remain ‘prediabetic’ or regress to normoglycemia. Thus, there is a growing need for innovative strategies to tackle diabetes at scale. The emergence of biomarker technologies has allowed more targeted therapeutic strategies for diabetes prevention (precision medicine), though largely confined to pharmacotherapy. Unlike most drugs, lifestyle interventions often have systemic health-enhancing effects. Thus, the pursuance of lifestyle precision medicine in diabetes seems rational. Herein, we review the literature on lifestyle interventions and diabetes prevention, describing the biological systems that can be characterized at scale in human populations, linking them to lifestyle in diabetes, and consider some of the challenges impeding the clinical translation of lifestyle precision medicine.

**Electronic supplementary material:**

The online version of this article (doi:10.1186/s12916-017-0938-x) contains supplementary material, which is available to authorized users.

## Background

Prediabetes, a state of chronically elevated but non-diabetic blood glucose, is one of the greatest healthcare challenges of our time, affecting more than 100 million people in Europe alone [1]. The term ‘prediabetes’ implies a state of glucose dysregulation destined to worsen, with various health agencies stressing that those affected are at high-risk of developing type 2 diabetes (T2D). However, annually, only 5–10% of people with prediabetes will develop the disease, with 10% regressing to normoglycemia [2]. Moreover, in those who remain prediabetic, there is scant evidence that elevated, non-diabetic blood glucose concentrations are causally related with clinical endpoints, and therefore intensively intervening solely on the basis of glycemia may not be cost-effective [3]. Nevertheless, if the subgroup(s) of people with prediabetes who progress to diabetes could be distinguished with reasonable certainty, the case for early intervention would be compelling given the largely intractable nature of the disease, primarily due to the manner in which the insulin secreting beta-cells deteriorate, the absence of accessible beta-cell restorative therapy, and the devastating consequences of diabetic complications.

T2D is primarily a disease of dysregulated carbohydrate metabolism, influenced by lipid storage and metabolism [4]. The disease occurs when the beta-cell insulin secretory capacity falls below the body’s requirements for insulin production, which are governed chiefly by the quantity of glucose entering the blood from the gut (dietary sources) or the liver (gluconeogenesis), and the rate at which glucose is consumed and metabolized in tissues and organs; this process is in turn governed by peripheral insulin sensitivity (influenced by intra-cellular lipid accumulation) and non-insulin-dependent glucose trafficking (attributable to exercise). Thus, it is unsurprising that the foundations of a westernized lifestyle (poor diet, physical inactivity, and obesity) are the core modifiable ‘exposures’ believed to cause T2D and the targets of most non-pharmacologic diabetes prevention programs [5]. However, even the most successful lifestyle interventions tend to delay (by roughly 3 years) rather than prevent the disease from occurring [6]. Moreover, there appears to be extensive between-individual variability in susceptibility to lifestyle risk factors [7] and response to therapies [8]. Thus, there is an unmet need for more effective lifestyle therapies.

The apparent inter-individual variability in susceptibility and response to lifestyle factors in diabetes has motivated the view that tailoring lifestyle interventions to a person’s biological characteristics could help optimize diabetes prevention and treatment. This appealing concept has motivated a burgeoning direct-to-consumer industry that promises to match diet and exercise products and services to the customer’s personal genomic (and other omic) characteristics. Indeed, the largest public-sector medical research agencies (NIH and European Union) are also investing heavily in research focused on this topic. Nevertheless, there is considerable skepticism about lifestyle precision medicine, particularly when commercialized [9]; even where biomarkers have been conclusively linked to susceptibility and response phenotypes, many barriers to clinical translation persist.

Many healthcare agencies advocate a lifestyle characterized by daily physical activity, a healthy diet, moderate alcohol consumption, maintenance of normal body weight, sound psychological health, and non-smoking [10, 11]. These recommendations are based on data from large epidemiological studies and randomized controlled trials (RCTs) indicating that these lifestyle factors lower risk of, or help prevent, T2D at a population level [11]. The most impactful risk factor and intervention target is excess body weight, as being obese (BMI 30–35 kg/m^2^) or very obese (BMI ≥ 35 kg/m^2^) conveys a 20- to 40-fold increased relative risk of T2D compared with being lean (BMI < 23 kg/m^2^) [12].

Herein, we review the concept of lifestyle precision medicine in T2D. Specifically, we summarize the published evidence on lifestyle risk factors and preventive interventions in T2D, briefly discussing the limitations of existing approaches focusing on lifestyle interventions for T2D prevention, and explore how harnessing biomarker technologies might help optimize these strategies. Furthermore, we describe evidence of differential response to lifestyle interventions based on unique genetic traits, as well as functional evidence of the biomolecular basis of these responses. Finally, we describe current omics technologies that can be applied to identify and stratify populations based on an individual’s unique genotype.

## Methods

### Literature search strategy

We reviewed the literature for evidence of modifiable lifestyle exposures that either raise or lower the risk of T2D. A detailed overview of each of the papers reviewed is given in Additional file 1: Table S1. The findings are summarized later in this review.

We searched the PubMed online database for papers using the search terms “(Cohort Studies[MeSH] OR Nested case cohort study[MeSH] OR Randomized Control Trial[MeSH]) AND (Lifestyle[MeSH] OR Environmental Exposures[MeSH]) AND (Blood glucose [MeSH] OR HbA1c [MeSH] OR Impaired fasting glucose OR Impaired glucose tolerance OR Dysglycemia OR Type 2 diabetes[MeSH] OR Glucose Intolerance[MeSH]) NOT review”. The search was restricted to human studies only and age 45 years and above without restriction on the year of publication.

The search retrieved information for 676 papers. After title scanning, 197 articles were selected as potentially relevant and their abstracts were reviewed, with 65 of these papers being relevant for full review. Additionally, further papers were identified through ancestral searches of bibliographies; therefore, a total of 75 papers [6, 12–85] were used to compile the evidence summary reported below.

## Review

### Published evidence of lifestyle in T2D risk and prevention

The review highlighted several well-conducted RCTs demonstrating the effect of lifestyle interventions in reducing the risk of T2D. In the Diabetes Prevention Program (DPP; n = 3234), an intensive lifestyle intervention was superior to a pharmacological intervention of metformin in reducing the incidence of diabetes over 2.8 years of follow-up compared to placebo [79]. The incidence of T2D was reduced by 58% (95% CI 48–66%) in the lifestyle group and by 31% (95% CI 17–43%) in the metformin group compared to the placebo group. Follow-up of the same cohort for a further 10 years showed persistent beneficial effects from lifestyle interventions, with T2D onset being delayed by approximately 4 and 2 years in the lifestyle and metformin groups, respectively, compared to placebo [6]. The Finnish Diabetes Prevention Study (DPS; n = 522) implemented a similar lifestyle protocol, which also conveyed a 58% (95% CI 30–70%) reduction in diabetes incidence [86]. Both protocols focused on overweight or obese (BMI > 25 kg/m^2^) participants with impaired glucose regulation, and promoted a 5–7% weight reduction, a total and saturated fat intake reduction, an increase in fiber intake, and regular exercise. Numerous other trials focusing on lifestyle interventions in a range of ethnic groups and study settings followed [20, 29, 58, 73], reporting reductions in diabetes incidence of comparable or lesser magnitude to the DPP and Finnish DPS trials. Long-term follow-up indicates that benefits of intensive lifestyle modification on diabetes incidence are sustained for up to 20 years [68].

Large prospective cohort studies have also reported robust associations between lifestyle exposures and T2D in diverse populations. For example, in the Kailuan prospective study (n = 50,656) [15], changes from the ideal cardiovascular health status score were inversely associated with risk of T2D over an average 3.8 years of follow-up. In the Finnish Twins Study (n = 20,487) [55], leisure-time physical activity reduced the risk of incident T2D in both monozygotic and dizygotic twins who were physically active compared to their sedentary siblings (HR 0.6, 95% CI 0.43–0.84, *P* = 0.003), even after factoring in familial risk and home environment. In the Strong Heart Study [64], among American Indians (n = 1651) followed for 10 years, high physical activity was associated with a reduced risk of T2D (OR 0.71, 95% CI 0.51–0.99 in the highest quartile compared to those who reported no physical activity), although the estimates were attenuated and became non-significant after adjusting for adiposity. A similar beneficial impact of physical activity in T2D incidence was observed in a European case-cohort study of nearly 30,000 adults [46], and in 68,000 female US health professionals [78].

Yates et al. [5] systematically reviewed the literature on RCTs testing the efficacy of diet and/or exercise interventions in the prevention of T2D. Of the eight trials reviewed, most involved a combined diet and exercise intervention. Compared with standard of care, the reduction in the risk of developing T2D attributed to the lifestyle interventions averaged 50% during the trial’s randomization phase. Long-term follow-up of the DPP Outcomes Study [6], the Finnish DPS [87], and the China Da Qing Diabetes Prevention Study [68], all indicated that the reduced risk of diabetes attributable to lifestyle intervention is sustainable for 10–20 years post-randomization. Although attempts have been made to parse out the relative contributions of diet and exercise in diabetes prevention, most lifestyle trials have not been designed for this purpose, and generally assess diet and exercise using self-report methods prone to respondent bias. However, Slentz et al. [88] recently reported that, within an exercise-only intervention trial, high volume moderate-intensity exercise (~18.2 km/week of walking) alone substantially reduced glucose tolerance in people at high risk of T2D, despite modest effects on body weight reduction (~2 kg). Diet-only interventions, such as that used in the PREDIMED trial focusing on Mediterranean-style diets [89], have yielded reductions in diabetes risk of approximately 50% compared with a control intervention.

RCTs are often considered the gold-standard in the hierarchy of causal evidence, as double-blind, placebo controlled trials are generally robust to confounding and reverse causality. However, in lifestyle intervention trials, masking treatment allocation from the participant and investigators is extremely challenging, which may result in compensatory behaviors that introduce bias and confounding – a rarely discussed caveat that affects the validity of data from lifestyle RCTs. Nevertheless, abundant epidemiological studies and clinical trials have implicated multiple lifestyle factors in the development of T2D.

Poorer social environments, within which fewer resources and opportunities exist to maintain healthy lifestyles, convey an increased risk for many diseases, including obesity and T2D [90]. Studies in twins suggest that the relationship between socioeconomic status and obesity may be modified by genetic variation [91]. Using data from the UK Biobank, Tyrrell et al. [92] studied the interaction of 66 established BMI-associated variants and 12 obesogenic lifestyle exposures in obesity; the authors extended previous discoveries of genetic interactions with physical activity [93, 94] and TV viewing [95], and identified a novel interaction with the Townsend Deprivation Index [96].

Evidence on the association between environmental exposures (e.g., to particulate matter and persistent organic pollutants) and T2D has yielded mixed results: some studies showed a statistically significant relationship between long-term exposure and risk of T2D [18], with higher risk attributed to traffic-specific pollution [33, 47], whereas others found no association [21]. Furthermore, one study reported an association of traffic-specific pollution exposure with T2D risk in women [51]. Exposure to arsenic [24, 81] and persistent organic pollutants was also significantly associated with T2D [19, 61], but no such association was seen for cadmium exposure [35, 44].

Coffee consumption has been associated with lower risk of dysglycemia in observational studies [48], yet recent Mendelian randomization analyses do not support a causal relationship [97, 98]. In the Adventist Health Studies [65] and the Women’s Health Study [75], consumption of red and processed meats was significantly associated with increased T2D risk. Intake of dairy products was not consistently related to T2D, cardiovascular disease, or all-cause mortality [38]. In the EPIC InterAct Study [99], dietary fiber consumption was associated with lower T2D incidence, though this was partially explained by body weight. While psychosocial health is an important risk factor, especially in the elderly [13], the relationship between depressive symptoms and dysglycemia may be mediated by other factors such as lifestyle [14]. Working overtime was significantly associated with risk of T2D among nurses [70] and among Japanese men working more than 50-hours overtime per month [83].

Although the literature on specific lifestyle exposures and T2D risk is extensive, all studies used to inform guidelines are based on the average estimated effects in the studied population, which would be acceptable if susceptibility to risk factors and response to preventive interventions were homogeneous. However, there is tremendous between-person variability in susceptibility and response to lifestyle exposures, which undermines the value of uniform recommendations. Indeed, it is estimated that the majority of people undergoing exercise interventions do not show an adequate response [8]. There are various reasons why there may be a lack of response to lifestyle interventions, many of which are irrelevant to individual biology; these factors are listed in Table 1. Ignoring these factors when estimating the likely impact of lifestyle precision medicine in T2D would substantially overestimate its value [100]. Nevertheless, harnessing genotypes and other omic variants to optimize lifestyle interventions for population subgroups may significantly impact individual and population-scale diabetes trajectories. Such approaches are especially appealing in prediabetes, where measures of glycemia alone are inadequate, and additional biomarkers are likely needed to predict or prevent progression to full-blown disease.

**Table 1:** Factors influencing response to lifestyle interventions

Factor	Definition
Behavioral compensation	In most cases, assignment to lifestyle interventions in clinical trials cannot be masked from the participants or investigators. This may prompt changes in behavior that are not the main objective of the trial and which differ by treatment arm, or may cause investigators to treat participants in the lifestyle and control arms differently. These sources of bias may underlie what appears to be variability in treatment response.
Regression to the mean	Trials that include only one outcome assessment, and which assess change in the outcome as the difference between the baseline and follow-up measure, are likely to be prone to regression dilution bias (or regression to the mean). This phenomenon occurs because most assessments are made with some degree of error, meaning that, in some participants, the change in the outcome will be underestimated and in others it will be overestimated. Where the outcome is assessed using a physical stress test (such as on a treadmill or bicycle ergometer), differences in effort at the beginning and end of the trial will also contribute to the apparent variability in treatment response. This problem could in principle be overcome in a randomized controlled trial by conditioning treatment response on response to the control intervention, although this is not conventionally done in studies of responders and non-responders, which generally focus only on intervention groups.
Adherence	Variability in the extent to which participants follow protocols in clinical trials (adherence) is likely to play a significant role in determining the extent to which an intervention appears to work. Although adherence is usually monitored in trials, monitoring adherence to lifestyle interventions is challenging, as the accurate and precise assessment of diet and exercise is notoriously difficult. The use of self-reported diet and/or exercise instruments to monitor adherence is likely to be insufficient in lifestyle trials, as participants in the active intervention arm may feel pressured to provide confirmatory responses to lifestyle questions.
Background heterogeneity in behaviors	Lifestyle interventions are often comprised of around 150 mins/week contact time, accounting for approximately 2% of all waking time. During the 98% non-contact time, participants’ behaviors are likely to vary considerably, influencing the extent to which the trial’s outcomes change.

Most contemporary studies of the interplay between genetic and lifestyle factors have focused on gene variants and lifestyle exposures previously associated with the disease of interest. Few of the exposures mentioned above have been studied in the context of gene–lifestyle interactions. A systematic search of the PubMed database (conducted on July 19, 2017; see Additional file 1: S1 for the search string) identified 30 original research articles and 13 review articles or commentaries. Of these, only seven publications focused on T2D as the outcome; additional ten relevant papers were identified through ancestral searches some of which are RCTs [101–106]. Others reported on prospective observational studies examining gene variant interactions with different diet components [107–110] and with physical activity [107, 111].

### Types of biomarkers

#### Genotypes

Two contrasting approaches have been used most in studies of genetics and lifestyle in complex disease traits. The dominant strategy has been the use of genome-wide association studies (GWAS), which leverage massively parallel genotyping technologies to interrogate variation across the genome, in a way that is agnostic to prior knowledge about genes, lifestyle, or disease. The second strategy involves studies focused on animal or human biology, through which genes and pathways have emerged as subsequent targets of epidemiological studies and/or clinical trials. The published findings from the latter are generally far less reproducible than those from studies using GWAS-based approaches.

#### Examples of GWAS-based studies connecting lifestyle and T2D

The use of large cohort collections within which genetic variation, self-reported measures of macronutrient intake, and other relevant factors are included have facilitated the discovery of variants associated with protein and carbohydrate intake. The CHARGE consortium reported robust associations between *FTO* variants and dietary protein intake in 177,000 adults [112], whereby each copy of the rs9939609 ‘A’ allele was associated with a mean of 0.08% (95% CI 0.06–0.10%, *P* = 2.4 × 10^–16^) higher protein intake; weak associations between the ‘A’ allele and lower total energy intake and lower dietary carbohydrate intake were also reported. The latter contradicts data from studies in children using objective measures of total energy intake, where the ‘A’ allele was associated with higher total energy intake [113].

There is compelling evidence that the hepatokine fibroblast growth factor 21, and variation at the gene that encodes it (*FGF21*), helps regulate energy homeostasis [114] and influences macronutrient and alcohol preference in animals and humans [115–118]. The FGF21 hormone is induced by metabolic stress, including ketogenesis and dietary carbohydrate consumption. FGF21 was initially suggested as a therapeutic agent for diabetes [114] by the pharmaceutical giant Eli Lilly and Co. Exogenous FGF21 infusion lowers blood glucose and triglyceride concentrations and improves pancreatic beta-cell function in db/db mice [119], yet endogenous FGF21 concentrations are positively associated with a range of dysmetabolic biomarkers, including blood glucose, insulin, and c-peptide concentrations in humans [120], which may reflect FGF21 resistance or other metabolic feedback processes. Nevertheless, despite its therapeutic potential, the FGF21 protein has rapid renal clearance [121], although the development of FGF21 analogues have helped circumvent this problem [122]. A phase I placebo-controlled clinical trial testing the effects of one such analogue (LY2405319) improved blood lipids and reduced body weight and fasting insulin, but showed no marked impact on blood glucose concentrations [123]. An alternative therapeutic approach might focus on dietary interventions designed to limit sugar-sweetened foods and beverages in carriers of susceptibility variants at *FGF21*. However, the extent to which each risk allele is associated with increased sugar consumption is negligible at an individual level [115], and is unlikely to be of any value for targeted dietary interventions in clinical practice.

There are many studies exploring interactions between gene variants and diet or exercise in T2D (see [124]), yet most are relatively small, some are cross-sectional, and few findings have been replicated. The largest, most comprehensive epidemiological analysis was performed by the InterAct consortium [107], where interactions between 58 established T2D variants and lifestyle factors were assessed in a nested case-cohort comprised of 12,403 incident cases of diabetes and 16,154 non-diabetics. In analyses focused on a gene-score, no statistically significant interactions were observed with physical activity or dietary habits assessed by a Mediterranean diet score; although several individual variants showed nominal evidence of gene–lifestyle interactions, none was significant after correction for multiple-testing. The largest and most comprehensive clinical trial analysis was performed in the DPP (n = 2843), which focused on a genetic risk score and its interactions with lifestyle intervention (vs. standard of care) [104]. There was no evidence of a gene–lifestyle interaction in diabetes incidence, but there was nominal evidence of an interaction in regression from impaired to normal glucose regulation. Follow-up analyses in the DPP trial, focusing on gene variants previously associated with insulin resistance, found that these did not influence the effects of lifestyle on insulin sensitivity after 1 year of intervention [125]. Overall, there is little robust evidence from epidemiological studies or clinical trials showing that variants previously associated with T2D or insulin resistance modify the effects of lifestyle in diabetes incidence.

#### Variation in the Tre-2/BUB2/cdc 1 domain family (TBC1D) genes as examples of biologic candidate genes connecting lifestyle and T2D

*TBC1D1* and *4* encode Rab-GTPase-activating proteins that regulate muscle glucose transport and fatty acid oxidation in response to insulin and exercise (see [126]). Abundant animal data implicate coding variation at *Tbc1d1* in exercise-related modulation of muscle glucose uptake and weight change, and in vitro perturbation of TBC1D1-transfected mouse myocytes by AICAR (an exercise mimetic) was shown to impact palmitate oxidation [127]; however, it remains unknown whether coding variation at *TBC1D1* in humans influences glucose and lipid metabolism. Evidence in humans of how *TBC1D4* variation impacts diabetes risk is more concrete. Homozygote carriers of the nonsense p.Arg684ter allele at *TBC1D4*, common within the Greenlandic Inuit but rare elsewhere, have a roughly 10-fold increased odds of T2D [128]. An exaggerated early insulin response was reported elsewhere in family members with acanthosis nigricans who carried a heterozygous substitution of thymine for cytosine at nucleotide position 1087 in exon 3, resulting in the substitution of a premature stop codon (TGA) for arginine (CGA) at codon 363 [129]. The mechanism appears to involve muscle-selective loss of the long isoform of TBC1D4, leading to a much reduced GLUT4-mediated insulin-stimulated glucose uptake into muscle and marked postprandial (but not fasting) hyperglycemia. What remains unknown is whether exercise-induced AS160 phosphorylation (the protein encoded by *TCB1D4*) and GLUT4 translocation (a key feature of insulin-dependent and non-insulin-dependent glucose transportation regulated by AS160 phosphorylation) also differ by *TBC1D4* genotypes in outbred populations and, if so, whether exercise might be sufficient to offset the impairments in GLUT4 sequestration attributable to TBC1D4 isoform restriction.

#### Transcripts, proteins, and epigenetic marks

The nuclear genome encodes biological processes that are necessary to maintain normal physiological function. The transcription and translation of genetic code can be perturbed by extrinsic and intrinsic environmental stimuli, and by chemical modifications of DNA (broadly termed ‘epigenetics’). In some instances, it may be that diet and exercise interact with epigenetic features, such that the physiological consequences of a lifestyle exposure are determined in part by the presence or absence of an epigenetic mark; in other cases, it may be that diet and exercise causes an epigenetic mark to emerge or disappear.

There is extensive literature on the effects of exercise or diet interventions on biomarkers of gene transcription and translation. The transcription (mRNA production) and translation (protein synthesis) of metabolic regulator genes, particularly those involved in mitochondrial biogenesis and mitochondrial function (e.g., *PPARGC1A*, *AMPK*, and *SIRT1*), have long been the focus of diet and exercise studies, particularly in the context of energy flux and substrate metabolism (see [130]). Although there are many intervention studies designed to test whether perturbation by diet or exercise affects molecular processes, most are not RCTs; this is an important limitation, as the absence of a control arm makes it impossible to determine that the intervention’s effects are not confounded by other unmeasured variables. This problem was highlighted in a study where many genes thought to be ‘exercise-induced’ were shown to change in both control and intervention arms, i.e., effects were not specific to exercise [131].

Most evidence linking lifestyle with changes in gene expression and epigenetic marks originates from cross-sectional cohorts. By contrast, RCTs focused on these issues are exceptionally rare. One of the few RCTs within which the effects of diet on metabolites and methylation marks have been studied is the LIPOGAIN trial [132]. In this double-blind, randomized, parallel-arm intervention trial, young adults (21–38 years; n = 41) were randomized to receive one of two types of high-energy content muffins, supplemental to their habitual diet, for 7 weeks; muffins contained either refined palm oil (rich in the major SFA palmitic acid (16:0)) or refined sunflower oil (rich in the major PUFA linoleic acid (18:2 n–6)). Abdominal subcutaneous adipose tissue was biopsied before and after the dietary intervention, liver fat was assessed using magnetic resonance imaging, and DNA and RNA were extracted for genome-wide methylation and mRNA analyses, respectively. Analysis of 37 of the 41 randomized participants explored changes in gene expression. Three genes, carbonic anhydrase 3, connective tissue growth factor, and aldehyde dehydrogenase 1 family member A1, were determined to be differentially expressed over time and between intervention arms. In a second report from a subset of the LIPOGAIN study (*n* = 31) [133], multiple changes in DNA methylation of individual genes and CpG sites were reported when focusing on both intervention arms combined or separately within intervention arms. In the within-arm analyses controlling for changes in cell composition and multiple testing, methylation levels changed at 309 sites within the SFA-rich muffin arm and at 4662 sites in the PUFA-rich muffin arm. However, whether expression and methylation were of greater or lesser magnitude between intervention arms was not reported, and there was no control arm.

#### Metabolites

Metabolites are intermediary compounds produced by naturally occurring enzyme-catalyzed reactions within cells, bearing the parent compound’s characteristics until fully degraded, and generated to control the rate of energy turnover and to perform other functions within cells. The measurement of metabolites in the body (usually in blood or urine) provides a read-out of specific metabolic processes. Examples of metabolites include amino acids within proteins, glucose molecules in glycogen, fatty acids within membrane lipids, and nucleotides in DNA [134].

Metabolomic profiling of diet and exercise signatures has yielded potentially useful tools to objectively assess these traits. For example, differential metabolomic profiles were determined using nuclear magnetic resonance spectroscopy in an observational study of twins discordant for physical activity, such that one twin of each pair reported no structured leisure-time physical activity, whereas the other reported more than 5 years regular leisure-time physical activity [135]. Twins who reported being physically active had lower serum concentrations of isoleucine, α1-acid glycoprotein, and glucose; moreover, the fatty acid profile was less saturated. In the INTERMAP UK cohort [136], ‘diet discriminative’ metabolomic profiles were generated within a randomized cross-over diet intervention trial by profiling urine samples taken following each 72-h diet intervention using proton nuclear magnetic resonance spectroscopy. In both studies, physical activity and diet profiles were validated by demonstrating associations between metabolomic signatures and disease outcomes known to be associated with diet and physical inactivity.

#### Microbiota

The human microbiota is comprised of four micro-organisms (bacteria, fungi, archaea, and viruses) that live in or on the body and serve multiple essential functions [137]. The ‘microbiome’ is the genetic material derived from all four micro-organisms taken from a given microbial ecosystem.

In a study of 800 young adults, Zeevi et al. [138] assessed glycemic responses to almost 50,000 meals. They found high between-participant variability in response and, using machine learning methods, they were able to derive and validate a prediction algorithm that harnessed data on the intestinal microbiome; they later designed personalized diet interventions and showed that these could be used to regulate glycemic excursions following meals and robust changes in the configuration of gut microbiota.

## Conclusions

Precision medicine research generally seeks to (1) elucidate the biology for the development of therapeutic targets or (2) identify biomarkers that can be used to inform health decisions and/or design new therapeutic strategies. In the latter, there are multiple contexts of use where biomarkers might play a key role in optimizing the design, timing, or delivery of lifestyle interventions (see [100]).

Determining whether discoveries in lifestyle precision medicine are likely to be of eventual clinical value first requires that the translational avenue be identified. In the development of anti-diabetic drugs, genetics has proven extremely valuable for the discovery of novel targets, reducing costs and improving drug development pipelines [139]. There are excellent examples where genetic screening is routinely undertaken for rare genetic disorders, such as phenylketonuria, where mutation carriers are prescribed special diets, or where genetics can provide insight into allergies or intolerances to specific foods or nutrients [140]. However, in T2D, genetics or other omic biomarkers are yet to meaningfully impact the optimization of lifestyle therapies, although two recent studies that used machine learning algorithms to interrogate complex data structures to predict individual response to foods [138, 141] highlight the possibilities ahead. There are numerous promising biomarkers that might eventually prove useful in this regard, as discussed above, but this requires the careful qualification of the biomarker in its proposed context of use.

For example, to predict the rate of T2D diagnosis, a biomarker’s predictive accuracy will need to be assessed, i.e., it should improve the accuracy of current prediction algorithms or enhance the reclassification of incident disease prediction. If the biomarker is to predict treatment response or side-effects, the assessment should be made in the context of an appropriately designed intervention study. The cost-effectiveness of lifestyle precision medicine strategies will also need to be demonstrated, as will the safety and scalability of such approaches. To date, no comprehensive attempts to do so in relation to biomarkers, lifestyle, and T2D have been described.

Curbing the global diabetes epidemic requires innovative approaches for its prevention. With the rapid development of biomarker technologies to characterize the etiology and pathogenesis of diabetes at scale, there are many ways in which lifestyle interventions could be optimized to help prevent T2D. However, major barriers to this vision include the assimilation and analysis of relevant, multi-omic biomarker data in specially designed lifestyle intervention trials. The analytical aspects alone are enormously challenging, as human biology is both complex and dynamic, but solutions are emerging [142]. The design and conduct of lifestyle trials for precision medicine also require innovative approaches to overcome the sources of bias and confounding that are difficult to circumvent when interventions cannot be masked. Nevertheless, recent advances in wearable technologies may help address these longstanding problems.

## Additional file

Additional file 1: Table S1. Studies used in the review and their main findings. (DOCX 40 kb)
