# Supplementary material for: Lifestyle precision medicine: the next generation in type 2 diabetes prevention?
Source: BMC Med. 2017 Sep 22;15:171. doi: 10.1186/s12916-017-0938-x (PMC5609030; doi:10.1186/s12916-017-0938-x)
Supplement: Additional file 1: Table S1. — Studies used in the review and their main findings. (DOCX 40 kb) [file 12916_2017_938_MOESM1_ESM.docx]

**Supplementary material.**

**Additional file 1: S1. Search string**

A systematic search of the PubMed database (conducted July 19, 2017), using the search terms “gene-lifestyle interaction” or “gene-exercise interaction” or “gene-diet interaction” or “gene-physical activity interaction” or “gene x lifestyle interaction” or “gene x exercise interaction” or “gene x diet interaction” or “gene x physical activity interaction” and “type 2 diabetes”, and restricting the search to “humans”, identified 30 original research articles and 13 review articles or commentaries.

**Additional file 1: Supplementary Table 1.** Studies used in the review and their main findings.

| Title | Author | Year | Type of study | Findings |
| --- | --- | --- | --- | --- |
| Healthy lifestyle and normal waist circumference are associated with a lower 5-year risk of type 2 diabetes in middle-aged and elderly individuals: Results from the healthy aging longitudinal study in Taiwan (HALST) | Chen et al. | 2017 | Prospective cohort | A healthy lifestyle and normal waist circumference were protective against T2D within 5 years, especially in those with impaired glucose tolerance or metabolic syndrome. Psychosocial health was an important lifestyle factor in the elderly. |
| Depressive symptoms and glycated hemoglobin A1c: a reciprocal relationship in a prospective cohort study | Schmitz et al. | 2016 | Prospective cohort | A “dynamic relationship” between depressive symptoms and HbA1c was observed, which may have been mediated by both lifestyle and cardio-metabolic factors. |
| Changes in ideal cardiovascular health status and risk of new-onset type 2 diabetes: The Kailua prospective study | Liu et al. | 2016 | Prospective cohort | Change in ideal cardiovascular health status was inversely associated with risk of T2D over a follow-up period of 3.8 years in both men and women. |
| Napping and the risk of type 2 diabetes: a population-based prospective study | Hublin et al. | 2016 | Prospective cohort | Frequent napping is associated with future risk of T2D. This association is largely explained by obesity. |
| Long-term exposure to fine particulate matter and incidence of type 2 diabetes mellitus in a cohort study: effects of total and traffic-specific air pollution | Weinmayr et al. | 2015 | Prospective cohort | Long-term exposure to total PM increases T2D risk in the general population, as does living close to a major road. Local traffic-specific PM was related to higher risks for T2D than total PM. |
| Persistent organic pollutants in young adults and changes in glucose related metabolism over a 23-year follow-up | Suarez-Lopez et al. | 2015 | Nested case control | Decades of exposure to persistent organic pollutants at background environmental levels was associated with dysglycemia independent of BMI, especially after the age of 50 years. |
| Long-term exposure to air pollution and type 2 diabetes mellitus in a multiethnic cohort | Park et al. | 2015 | prospective cohort | In this large, community-based, multiethnic prospective study, long-term exposure to atmospheric PM and nitrogen oxides was significantly associated with prevalent T2D at baseline. There was no significant association with the development of T2D over the follow-up period (9 years), though a significant effect modification by sex of the association between nitrogen oxides and T2D was observed. |
| Accumulation of major life events in childhood and adult life and risk of type 2 diabetes mellitus | Masters Pedersen et al. | 2015 | Prospective cohort | The cumulative effect of major life events in childhood and adult (personal and work) life is a risk factor for developing T2D. |
| Birth weight and later life adherence to unhealthy lifestyles in predicting type 2 diabetes: prospective cohort study | Li et al. | 2015 | Prospective cohort | Low birth weight and unhealthy lifestyle in adult life additively interact to increase the risk of T2D, with this interaction accounting for about 18% of the population-attributable fraction. |
| Arsenic exposure, arsenic metabolism, and incident diabetes in the strong heart study | Kuo et al. | 2015 | Prospective cohort | Markers of arsenic metabolism, especially low levels of monomethylarsonate, were associated with an increase in the risk of incident T2D. The researchers suggested that more research is needed on this metabolic connection. |
| The effect of lifestyle intervention and metformin on preventing or delaying diabetes among women with and without gestational diabetes: The Diabetes Prevention Program outcomes study 10-year follow-up | Aroda et al. | 2015 | RCT | Lifestyle and metformin interventions were effective in delaying T2D in women with a history of gestational diabetes mellitus during a 10-year follow-up period. Lifestyle, but not metformin, was effective in women without a history of gestational diabetes mellitus. |
| Long-term effects of lifestyle intervention or metformin on diabetes development and microvascular complications over 15-year follow-up: The Diabetes Prevention Program Outcomes Study | Nathan et al. | 2015 | RCT | Over the follow-up time, lifestyle or metformin interventions significantly reduced incidence of T2D. No overall differences were observed in aggregate microvascular outcome, but microvascular complications were lower in those who did not develop T2D. |
| Television viewing time and risk of incident diabetes mellitus: the English Longitudinal Study of Ageing | Smith et al. | 2014 | Prospective cohort | A high television time with physical inactivity at baseline was associated with increased risk of T2D. |
| Physical activity, weight status, diabetes and dementia: a 34-year follow-up of the population study of women in Gothenburg | Mehlig et al. | 2014 | Prospective cohort | Low physical activity and obesity were associated with increased risk of both T2D and dementia. |
| Modifying effect of obesity on the association between sitting and incident diabetes in post-menopausal women | Manini et al. | 2014 | Prospective cohort | The duration of sitting was independently associated with T2D risk elevation in obese women. |
| 10-year incidence of diabetes and associated risk factors in Greece: the ATTICA study (2002-2012) | Koloverou et al. | 2014 | Prospective cohort | Physical activity was associated with low risk of T2D, ameliorating the deleterious effects of family history and impaired glucose tolerance. Waist-to-height ratio was the best predictor of T2D among anthropometric measures. |
| Residential traffic and incidence of Type 2 diabetes: the German Health Interview and Examination Surveys | Heidemann et al. | 2014 | Prospective cohort | Persons exposed to high traffic had a two-fold higher increase in risk of T2D. |
| Long-term air pollution exposure and diabetes in a population-based Swiss cohort | Eze et al. | 2014 | Cross-sectional | Long-term exposure to PM and nitrogen oxides at concentrations below air quality guidelines was positively associated with prevalent T2D. |
| Cadmium exposure and incidence of diabetes mellitus - results from the Malmo Diet and Cancer study | Borne et al. | 2014 | Prospective cohort | High levels of cadmium in the blood were not associated with elevated T2D incidence. |
| Lifestyle and the risk of diabetes mellitus in a Japanese population | Tatsumi et al. | 2013 | Prospective cohort | Healthy lifestyle, assessed using a modified Breslow’s lifestyle index (covers smoking, alcohol intake, physical activity, sleep duration, BMI and day’s meal frequency including breakfast), was associated with lower incidence of diabetes in men and women in a Japanese cohort followed for 10 years. |
| Dairy product intake in relation to glucose regulation indices and risk of type 2 diabetes | Struijk et al. | 2013 | Prospective cohort | Total dairy or any subgroup was not significantly associated with incidence of T2D, despite modest benefits of fermented dairy and cheese on glucose levels. |
| Consumption of dairy products and associations with incident diabetes, CHD and mortality in the Whitehall II study | Soedamah-Muthu et al. | 2013 | Prospective cohort | Total dairy intake or groups of dairy products was not consistently related with incidence of T2D, coronary heart disease, or all-cause mortality. |
| The association of maximum body weight on the development of type 2 diabetes and microvascular complications: MAXWEL study | Lim et al. | 2013 | Prospective cohort | Rapid and high weight gain was associated with early onset of T2D, dysglycemia and diabetic complications especially nephropathy and retinopathy. |
| Association between modifiable lifestyle factors and residual lifetime risk of diabetes | Djousse et al. | 2013 | Prospective cohort | Healthy lifestyle (indicated by 5 lifestyle factors: regular exercise, normal body weight, healthy diet, moderate drinking, and non-smoking) was associated with a lower residual lifetime risk of T2D in both men and women in two different cohorts. Looking at the factors individually, adiposity had the highest association with risk of T2D. |
| Risk of incident diabetes in relation to long-term exposure to fine particulate matter in Ontario, Canada | Chen et al. | 2013 | Prospective cohort | Long-term exposure to atmospheric PM may contribute to the development of T2D. |
| Caffeinated and caffeine-free beverages and risk of type 2 diabetes | Bhupathiraju et al. | 2013 | Prospective cohort | Irrespective of the caffeine content, intake of sugar sweetened beverages was associated with a higher risk of T2D, and coffee intake was associated with a lower risk of T2D. |
| Cadmium exposure in relation to insulin production, insulin sensitivity and type 2 diabetes: a cross-sectional and prospective study in women | Barregard et al. | 2013 | mixed: cross-sectional with prospective component | Cadmium exposure was not associated with elevated T2D risk of impaired glucose tolerance. |
| Effects of healthy dietary pattern and other lifestyle factors on incidence of diabetes in a rural Japanese population | Morimoto et al. | 2012 | Prospective cohort | A healthy diet was associated with low risk for T2D in a rural Japanese population, especially in those who had a regular healthy diet, regular exercise and those who were not current smokers. |
| Physical activity reduces the risk of incident type 2 diabetes in general and in abdominally lean and obese men and women: the EPIC-InterAct Study | Ekelund et al. | 2012 | Case cohort | Physical activity, which included both commuting and leisure-time physical activity, was associated with a reduction in the risk of T2D in men and women, independent of adiposity. |
| Air pollution and incidence of hypertension and diabetes mellitus in black women living in Los Angeles | Coogan et al. | 2012 | Prospective cohort | Exposure to polluted air, especially traffic-related pollutants, was associated with increased risk of T2D and hypertension. |
| Coffee consumption and the incidence of type 2 diabetes in men and women with normal glucose tolerance: The Strong Heart Study | Zhang | 2011 | Prospective cohort | Consumption of high amounts of coffee was associated with a reduced risk of degenerating to dysglycemia during an average 7.6 years of follow-up. |
| Are particulate matter exposures associated with risk of type 2 diabetes? | Puett et al. | 2011 | Prospective cohort | Over 12 months, there was no strong evidence for the association between exposure to PM and incident T2D, but there was an association between T2D with traffic exposure (distance to road) in women. |
| Objectively measured physical activity and the subsequent risk of incident dysglycemia: The Australian Diabetes, Obesity and Lifestyle Study (AusDiab) | Ponsonby et al. | 2011 | Prospective cohort | High physical activity (a higher rate of daily steps) was associated with a reduced risk of incident dysglycemia among community-dwelling adults, independent of adiposity. |
| A prospective study investigating the association between environmental tobacco smoke exposure and the incidence of type 2 diabetes in never smokers | Ko et al. | 2011 | Prospective cohort | The study suggests that ETS (secondary smoking) exposure is a significant risk factor for the development of T2D with a dose-response relationship. |
| Changes in alcohol consumption and subsequent risk of type 2 diabetes in men | Joosten et al. | 2011 | Nested case control | Increases in alcohol consumption over time were associated with lower risk of T2D among initially rare and light drinkers. This lower risk was evident within a 4-year period following increased alcohol intake. |
| Leisure-time physical activity and type 2 diabetes during a 28-year follow-up in twins | Waller et al. | 2010 | Prospective cohort | Over 28 years of follow-up, leisure time physical activity was associated with reduced risk of T2D in twins (both monozygotic and dizygotic) when home environment and genetic predisposition were controlled for. Additional controlling for adiposity (BMI) did not change the association. |
| Association of passive and active smoking with incident type 2 diabetes mellitus in the elderly population: the KORA S4/F4 cohort study | Kowall et al. | 2010 | Prospective cohort | Both active and passive smoking were associated with increased risk of T2D. |
| Combined effect of alcohol consumption and lifestyle behaviors on risk of type 2 diabetes | Joosten et al. | 2010 | Prospective cohort | In subjects already at lower risk of T2D on the basis of multiple low-risk lifestyle behaviors, moderate alcohol consumption was associated with an approximately 40% lower risk compared with abstention. |
| Calcium, vitamin D and dairy intake in relation to type 2 diabetes risk in a Japanese cohort | Kirii et al. | 2009 | Prospective cohort | Overall, calcium intake was not associated with a significantly lower risk of T2D, but in those who had higher intake of vitamin D, calcium intake was associated with lower risk of T2D. Intake of dairy food products was significantly associated with a lower risk of T2D in women only. There is need for further research into the joint action of these nutrients, as calcium and vitamin D may not be independently associated with risk of T2D. |
| Physical activity and incident diabetes in American Indians: The Strong Heart Study | Fretts et al. | 2009 | Prospective cohort | Physical activity was significantly associated with a low risk of T2D in American Indians. |
| Meats, processed meats, obesity, weight gain and occurrence of diabetes among adults: findings from Adventist Health Studies | Vang et al. | 2008 | Prospective cohort | Consumption of red meats and processed meats (especially salted fish and frankfurters) was significantly associated with an increased risk of T2D. |
| Dietary patterns and risk of incident type 2 diabetes in the Multi-Ethnic Study of Atherosclerosis (MESA) | Nettleton et al. | 2008 | Prospective cohort | Over 5 years of follow-up, healthy diet (based on a low risk diet score) was associated with a lower risk of T2D in a multi-ethnic cohort, irrespective of ethnicity. |
| Incident diabetes and pesticide exposure among licensed pesticide applicators: Agricultural Health Study, 1993-2003 | Montgomery et al. | 2008 | Prospective cohort | Chlorine and some organophosphate-based pesticides were significantly related to the risk of T2D. |
| Prospective study of coffee and tea consumption in relation to risk of type 2 diabetes mellitus among men and women: the Whitehall II study | Hamer et al. | 2008 | Prospective cohort | Coffee intake was associated with risk of T2D at baseline. After follow-up, only combined coffee and tea intake was significantly associated with reduced risk of T2D. |
| Work characteristics and incidence of type 2 diabetes in women | Kroenke et al. | 2007 | Prospective cohort | Working overtime among young and middle-age nurses was significantly associated with increase in the risk of T2D. |
| Polybrominated biphenyls, polychlorinated biphenyls, body weight, and incidence of adult-onset diabetes mellitus | Vasiliu et al. | 2006 | Prospective cohort | There was no association between polybrominated biphenyl serum levels and incident T2D, but polychlorinated biphenyl serum levels were associated with risk of incident T2D in women. |
| Body mass index history and risk of type 2 diabetes: results from the European Prospective Investigation into Cancer and Nutrition (EPIC)-Potsdam Study | Schienkiewitz et al. | 2006 | Prospective cohort | An increase in weight in early adulthood (20–40 years) was associated with a higher risk of T2D than an increase in weight later in life (40–50 years). |
| Dairy consumption and risk of type 2 diabetes mellitus in men: a prospective study | Choi et al. | 2005 | Prospective cohort | Consumption of dairy products, especially low fat ones, was significantly associated with reduced risk of T2D in men. |
| A prospective study of red meat consumption and type 2 diabetes in middle-aged and elderly women: the women's health study | Song et al. | 2004 | Prospective cohort | Consumption of high amounts of red and processed meats (assessed in both frequency and quintiles of intake), was associated with a high risk of incident T2D in women. |
| Physical activity, obesity, and the incidence of type 2 diabetes in a high-risk population | Kriska et al. | 2003 | Prospective cohort | Incidence of T2D was lower in those who were more physically active compared to those who were less active. |
| Television watching and other sedentary behaviors in relation to risk of obesity and type 2 diabetes mellitus in women | Hu et al. | 2003 | Prospective cohort | Time spent on sedentary activities was significantly associated with increased risk of T2D and obesity, while increase in physical activity was inversely associated with T2D and obesity in women. |
| Diet, lifestyle, and the risk of type 2 diabetes mellitus in women | Hu et al. | 2001 | Prospective cohort | Using a set of lifestyle factors (BMI, diet, physical activity, smoking, and alcohol consumption), participants in the lower risk category had lower incidence of T2D compared to the higher risk category. A high BMI was the single most important factor associated with a very high risk of T2D in this population of women. |
| Long-term arsenic exposure and incidence of non-insulin-dependent diabetes mellitus: a cohort study in arseniasis-hyperendemic villages in Taiwan | Tseng et al. | 2000 | Prospective cohort | A high cumulative exposure to arsenic was significantly associated with risk of T2D in a dose-dependent way. Risk was highest in high cumulative exposure group compared to low exposure group. |
| Birthweight and the risk for type 2 diabetes mellitus in adult women | Rich-Edwards et al. | 1999 | Prospective cohort | Women who had a low birthweight had increased risk of T2D compared with their counterparts who had median or higher birth weight. This association remained significant even after adjustment for adulthood adiposity. |
| Overtime, psychosocial working conditions, and occurrence of non-insulin dependent diabetes mellitus in Japanese men | Kawakami et al. | 1999 | Prospective cohort | Among Japanese men, working more than 50 hours per month overtime was associated with increase in the risk of T2D compared to 0–25 hours per month. Workers who used new production technology at work also had a higher risk of T2D compared to those who did not. |
| Sex difference in lifestyle factors predictive of diabetes in Mexican-Americans | Monterrosa et al. | 1995 | Prospective cohort | In men, physical activity was significantly associated with a decreased risk of T2D, while an increase in alcohol consumption was associated with increased risk; BMI had a non-significant association. In women, a high BMI was significantly associated with increased risk of T2D, but association with physical activity was not significant. However, the low number of participants in this study may have contributed to these different findings between men and women. |
| Mediterranean diet and type 2 diabetes risk in the European Prospective Investigation into Cancer and Nutrition (EPIC) study: The Inter-Act project | Romaguera et al. | 2011 | case cohort | Adherence to a Mediterranean diet (score-based) reduced the risk of T2D with high scoring participants having a lower risk compared to those with a low score. |
| Exposure to p,p'-DDE: a risk factor for type 2 diabetes | Rignell-Hydbom et al. | 2009 | nested case control | Women who had high levels of the persistent organochlorine pollutant exposure biomarker, 1,1- dichloro-2,2-bis (p-chlorophenyl)-ethylene, were at a higher risk of T2D in later life compared to those who had low levels. |
| Effects of the lifestyle intervention program GLICEMIA in people at risk for type 2 diabetes: a cluster-randomized controlled trial | Schmiedel et al. | 2015 | RCT | A lifestyle intervention (3 individual and 5 group counseling sessions on diet, physical activity, and overall information on T2D and its risk factors and healthy living) improved the diabetic risk score (FINDRISC) in the intervention group compared to controls. |
| Reduction in incidence of type 2 diabetes by lifestyle intervention in a middle eastern community | Harati et al. | 2010 | RCT | The incidence of T2D was reduced in the lifestyle intervention group (physical activity, weight loss, smoking cessation, diet) compared to controls. |
| 10-year follow-up of diabetes incidence and weight loss in the Diabetes Prevention Program Outcomes Study | Knowler et al. | 2009 | RCT | Effects of ILS and metformin interventions in the prevention of T2D persisted for at least 10 years, with ILS having the highest impact in reduction of T2D compared to placebo. ILS delayed onset of T2D by 4 years while metformin delayed it by 2 and ILS had the best rates of regression to normoglycemia (23%) after 10 years. |
| Dietary fiber and incidence of type 2 diabetes in eight European countries: the EPIC-InterAct Study and a meta-analysis of prospective studies | Kuijsten et al. | 2015 | Mixed: longitudinal (nested case cohort) and meta-analysis | Participants who reported high consumption of dietary fiber had low incidence of T2D compared to low consumers. This association was partially explained by body weight. |
| Television watching and incident diabetes: findings from the European Prospective Investigation into Cancer and Nutrition-Potsdam Study | Ford et al. | 2010 | Prospective cohort | Participants who had more hours per week of watching television were at a higher risk of T2D compared to those with fewer hours. However, this association was attenuated when adjusted for BMI. |
| Physical activity and television watching in relation to risk of type 2 diabetes: The Black Women's Health Study | Krishnan et al. | 2009 | Prospective cohort | In a population of African-American women followed for 10 years, vigorous physical activity was associated with low risk of T2D, while TV time (sedentary time) was positively associated with risk of T2D, independent of BMI |
| Physical activity and television watching in relation to risk for type 2 diabetes mellitus in men | Hu et al. | 2001 | Prospective cohort | High physical activity was related to reduced risk of T2D in men, while sedentary time (assessed as TV-time) was associated with increased risk in men. |
| Reduction in the incidence of type 2 diabetes with lifestyle intervention or metformin | Knowler et al. | 2002 | RCT | An intense lifestyle intervention was associated with the highest reduction in risk of T2D (and regression to normoglycemia) than metformin compared to placebo in men and women, who had impaired glucose tolerance. |
| Prevention of T2D mellitus by changes in lifestyle among subjects with impaired glucose tolerance | Tuomilehto et al. | 2001 | RCT | Lifestyle changes were associated with reduced risk of T2D (and improved glycemia biomarkers, clinical and anthropometric parameters) in the intervention group compared to controls. There was a strong inverse correlation between achieving lifestyle goals and the risk of T2D. |
| Effects of diet and exercise in preventing NIDDM in people with impaired glucose tolerance | Pan et al. | 1997 | RCT | Diet and exercise interventions reduced the risk of incident diabetes in participants randomized to the intervention arms compared to controls over 6 years of follow-up. |
| The long-term effect of lifestyle interventions to prevent diabetes in the china DaQing diabetes prevention study: A 20-year follow-up study | Li et al. | 2008 | RCT | The effects of exercise and diet interventions persisted over 20 years in a Chinese population of men and women, who had impaired glucose tolerance at baseline. The intervention group had 43% lower incidence than controls and the risk of progressing to diabetes remained high (93%) without any intervention. |
| Association of urinary metals levels with type 2 diabetes risk in coke oven workers | Liu et al. | 2016 | Cross-sectional | Urinary levels of copper, zinc, arsenic, selenium, molybdenum, and cadmium were significantly associated with high risk of T2D in coke oven workers. |
| Health risk factor modification predicts incidence of diabetes in an employee population: results of an 8-year longitudinal cohort study | Rolando et al. | 2013 | prospective cohort | Changes in BMI from high to low were associated with decreased risk of T2D among university employees, while increase in BMI increased the risk of T2D over the follow-up time. |
| Dietary patterns and the risk of type 2 diabetes in overweight and obese individuals | Bauer et al. | 2013 | Prospective cohort | Among overweight and physically less active persons, unhealthy diet pattern (scored based on low in fruits and vegetables and high in snacks and soft drinks mainly) was associated with a high risk of T2D. |
| Improvement in diet habits, independent of physical activity helps to reduce incident diabetes among prediabetic Asian Indian men | Ram et al. | 2014 | RCT | Achievement of lifestyle improvement goals (lowering BMI, decrease carbohydrates, oils and food portion, increase physical activity) was associated with decreased risk of T2D in the intervention group compared to controls. |
| Prevention of type 2 diabetes in a primary healthcare setting: three-year results of lifestyle intervention in Japanese subjects with impaired glucose tolerance | Sakane et al. | 2011 | RCT | A lifestyle intervention of increase in energy expenditure and lowering body weight was associated with a decrease in the risk of T2D among Japanese subjects with impaired glucose tolerance. |
| The Indian Diabetes Prevention Program shows that lifestyle modification and metformin prevent type 2 diabetes in Asian Indian subjects with impaired glucose tolerance (IDPP-1) | Ramachandranet al. | 2006 | RCT | In this DPP replica study, both exercise and metformin were associated with reduction in T2D incidence, with lifestyle having the highest impact. There was no added benefit of combining the interventions. |
| Prevention of diabetes mellitus in subjects with impaired glucose tolerance in the Finnish Diabetes Prevention Study: results from a randomized clinical trial | Lindstrom | 2003 | RCT | Compared to the control group, participants with impaired glucose tolerance randomized to receive a lifestyle intervention had lower cumulative incidence of T2D and this was inversely related to the number of lifestyle intervention goals achieved. |

*BMI* body mass index, *DPP* Diabetes Prevention Program, *ILS* intense lifestyle, *PM* particulate matter, *RCT* randomized controlled trial, *T2D* type 2 diabetes
